# Identification of ALYREF in pan cancer as a novel cancer prognostic biomarker and potential regulatory mechanism in gastric cancer

**DOI:** 10.1038/s41598-024-56895-5

**Published:** 2024-03-15

**Authors:** Yujie Yuan, Yiyang Fan, Wenqing Tang, Hui Sun, Jinghan Sun, Hongmeng Su, Hong Fan

**Affiliations:** 1 The Key Laboratory of Developmental Genes and Human Diseases, Department of Medical Genetics and Developmental Biology, School of Medicine, Ministry of Education, Southeast University, Nanjing, 210009 China; 2 Department of Biostatistics, University of North Carolina at Chapel Hill, Chapel Hill, NC 27599 USA; 3 School of Life Science and Technology, Southeast University, Nanjing, 210096 China

**Keywords:** Cell biology, Gastroenterology

## Abstract

ALYREF is considered as a specific mRNA m5C-binding protein which recognizes m5C sites in RNA and facilitates the export of RNA from the nucleus to the cytoplasm. Expressed in various tissues and highly involved in the transcriptional regulation, ALYREF has the potential to become a novel diagnostic marker and therapeutic target for cancer patients. However, few studies focused on its function during carcinogenesis and progress. In order to explore the role of ALYREF on tumorigenesis, TCGA and GTEx databases were used to investigate the relationship of ALYREF to pan-cancer. We found that ALYREF was highly expressed in majority of cancer types and that elevated expression level was positively associated with poor prognosis in many cancers. GO and KEGG analysis showed that ALYREF to be essential in regulating the cell cycle and gene mismatch repair in tumor progression. The correlation analysis of tumor heterogeneity indicated that ALYREF could be specially correlated to the tumor stemness in stomach adenocarcinoma (STAD). Furthermore, we investigate the potential function of ALYREF on gastric carcinogenesis. Prognostic analysis of different molecular subtypes of gastric cancer (GC) unfolded that high ALYREF expression leads to poor prognosis in certain subtypes of GC. Finally, enrichment analysis revealed that ALYREF-related genes possess the function of regulating cell cycle and apoptosis that cause further influences in GC tumor progression. For further verification, we knocked down the expression of ALYREF by siRNA in GC cell line AGS. Knockdown of ALYREF distinctly contributed to inhibition of GC cell proliferation. Moreover, it is observed that knocked-down of ALYREF induced AGS cells arrested in G1 phase and increased cell apoptosis. Our findings highlighted the essential function of ALYREF in tumorigenesis and revealed the specific contribution of ALYREF to gastric carcinogenesis through pan-cancer analysis and biological experiments.

## Introduction

Cancer remains the world’s leading cause of death with increased morbidity and mortality, although the clinical treatments including surgery operation, chemotherapy, radiotherapy, immunotherapy have improved the survival outcome of tumor patients^1^. Therefore, there is an urgent need for a novel diagnostic marker and therapeutic target molecule for cancer patients. In the past few years, extended studies in RNA biology have disclosed RNA methylation involved in carcinogenesis by promoting regulatory RNA function^2–4^. Increasing evidence supported that 5-methylcytosine in RNA is associated with multifarious cellular processes during tumorigenesis, such as cell proliferation, cell migration and tumor progression^5^. In m5C-dependent regulation, writer NOP2/Sun RNA Methyltransferase 2 (NSUN2) plays crucial roles in tumor progression^6,7^, Aly/REF export factor (ALYREF) considered as a reader of mRNA m5C facilitates the export of RNA from the nucleus to the cytoplasm^8,9^.

ALYREF, localized on chromosome band 17q25.3 and functions as a molecular chaperone, is involved in the transcriptional regulation^10,11^. ALYREF is expressed in the esophagus, kidney, stomach, liver, lung, colon, thyroid, and 20 other tissues^12^. To be of interest, ALYREF was involved in transcription elongation, genome stability^13,14^, RNA export from nucleus, and mRNA processing^9^. More and more evidence testified that ALYREF plays an important role in diversified diseases, especially cancers. In recent years, ALYREF is confirmed the oncogenic roles in hepatocellular carcinoma (HCC) development^8^ and considered as promising prognosis prediction^15^. However, up to date, the limited related studies of ALYREF has obstructed the understanding of its function during carcinogenesis and progress.

In the present study, we comprehensively analyzed the role of ALYREF expression, prognosis, genomic heterogeneity mutation and pathways in a series of tumors based on The Cancer Genome Atlas (TCGA) and multiple public databases. We investigated the biological process and potential pathways corelated to ALYREF by Gene Ontology (GO), Kyoto Encyclopedia of Genes and Genomes (KEGG). We further analyzed the co-expressed genes as well as mutations in all patients with gastric cancer and their correlation with ALYREF level. In vitro, knockdown of ALYREF suppressed the cell proliferation and foci formation in gastric cancer indicating the potential carcinogenic function of ALYREF on carcinogenesis. Our results implied that ALYREF could be considered as a promising prognostic biomarker and therapeutic target for gastric cancer.

## Results

### Differential expression profiles of ALYREF and correlation with prognosis in pan-cancer

**Figure 1:**
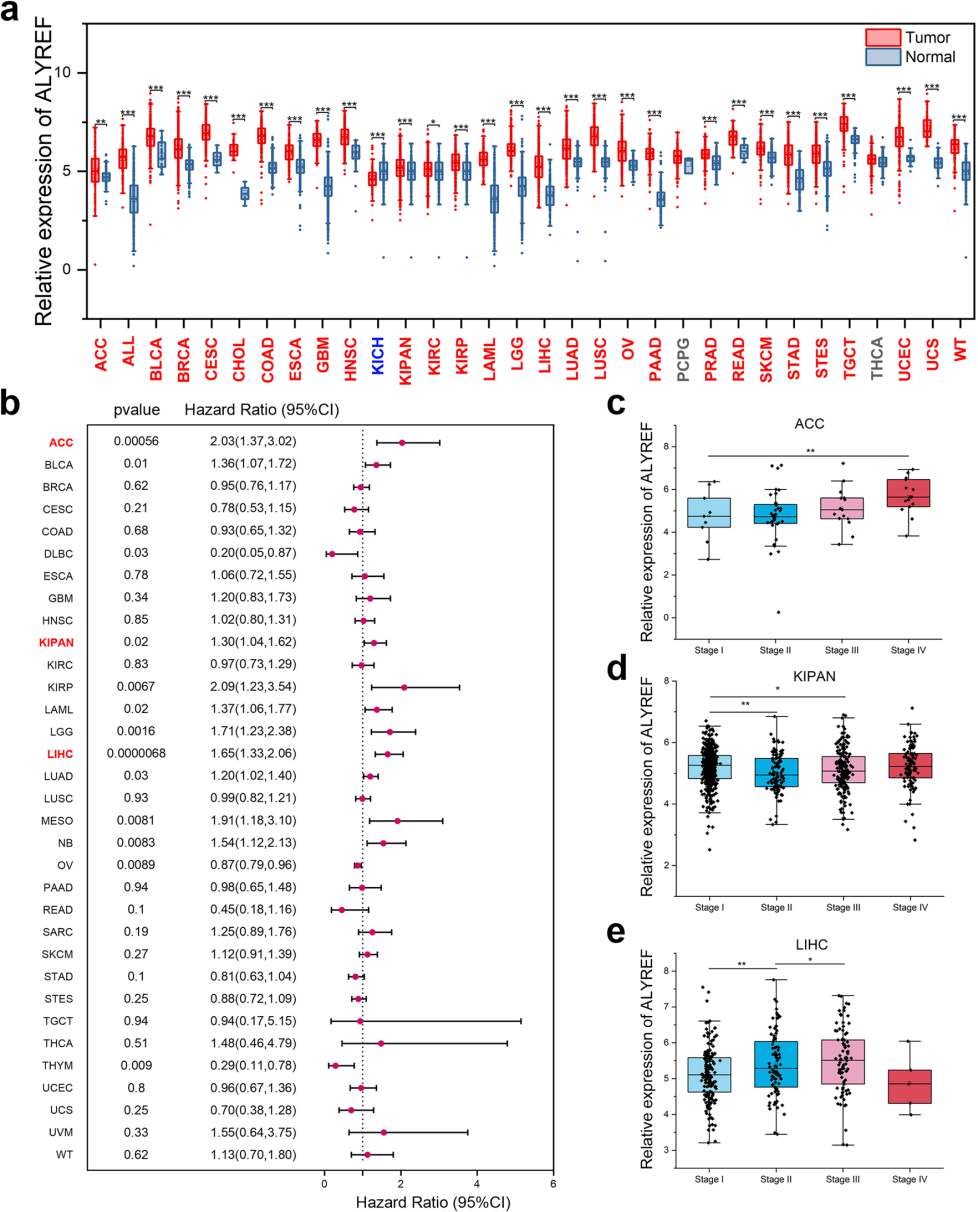
The gene expression of ALYREF and prognosis analysis in various cancer types. (**a**) Expression level of ALYREF in different types of human cancers based on TCGA and GTEx. (Cancer species with up-regulated expression of ALYREF in tumor tissues were highlighted in bold red font, down-regulated expression of ALYREF in tumor tissues were highlighted in bold blue font, and those with no significant difference between normal and tumor tissues were highlighted in bold gray font.) (**b**) Forest plot of the association between ALYREF and overall survival (OS) in various cancer types. (Cancer species mentioned below were highlighted in bold red font.) ALYREF expression level in ACC (**c**), KIPAN (**d**), and LIHC (**e**) at each stage (*p-value < 0.05, **p-value < 0.01, ***p-value < 0.001).

Expression level and pattern analysis based on TCGA and GTEx database revealed that the expression level of ALYREF were significantly increased in majority of cancers. Among the 32 types of cancer, 29 showed an increased level of ALYREF in tumors tissues than in normal tissues, including STAD, liver hepatocellular carcinoma (LIHC), and Pan-kidney cohort (KIPAN) (Fig. 1a). Thyroid carcinoma (THCA) and pheochromocytoma and paraganglioma (PCPG) were the only two cancers without an apparent difference in ALYREF expression. Kidney Chromophobe (KICH) displayed a decreased expression level of ALYREF in tumors than in normal tissue (p < 0.001). Realizing the effect of ALYREF level on tumors, we continued to analyze the relationships between the prognostic values of tumors and ALYREF level. According to Fig. 1b, the expression of ALYREF was closely related to the prognostic value of various tumors. Several cancer types showed worse survival outcome with higher ALYREF level. We chose the three cancer types with the greatest significance level, ACC, KIPAN, and LIHC and studied the correlation between ALYREF gene expression and cancer stages. All three cases showed a strong correlation (Fig. 1c–e). These data showed that ALYREF level were closely related to the development of several tumor types.

### ALYREF-related pathways in pan-cancer

**Figure 2:**
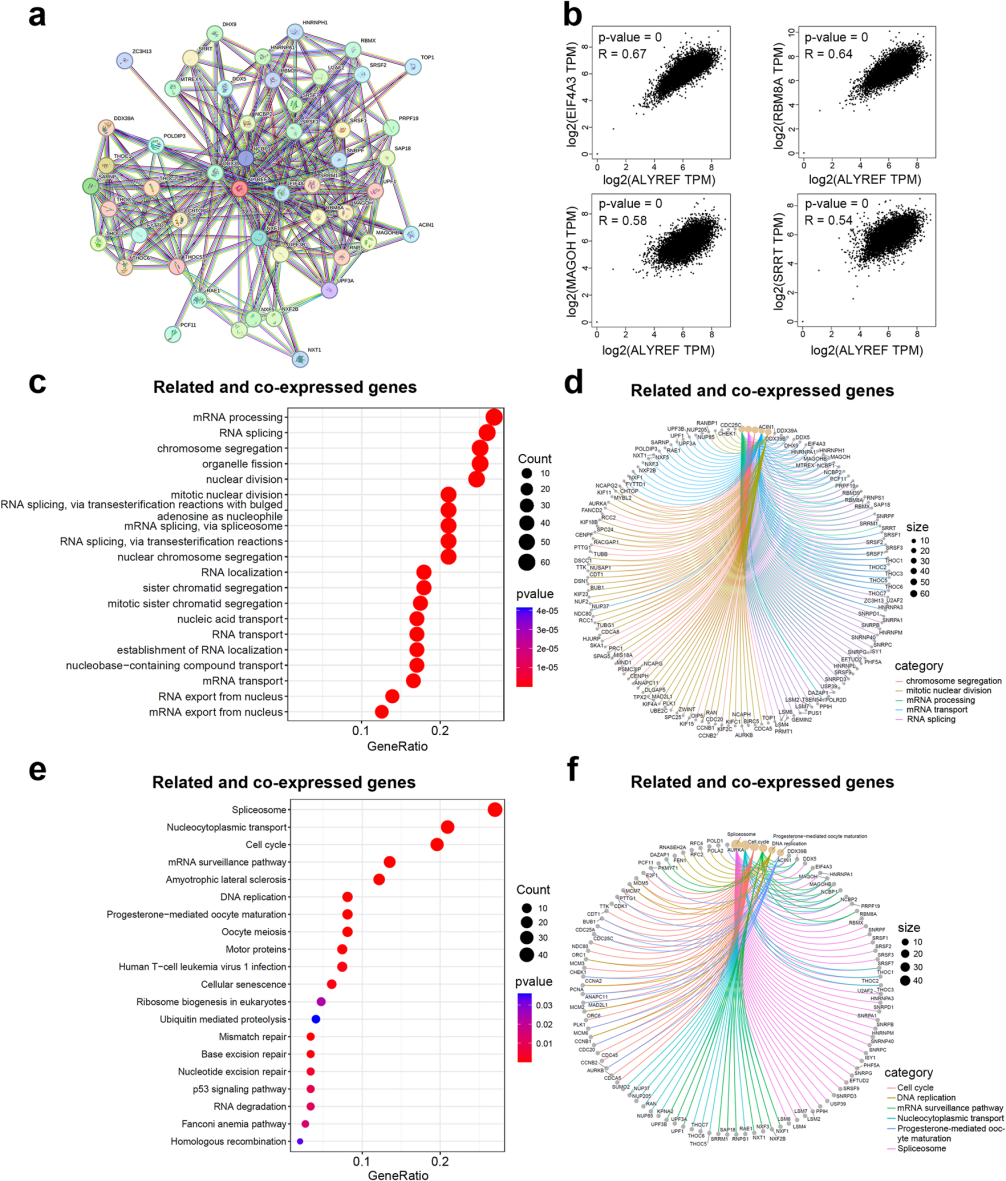
Biological function analysis of ALYREF-related genes. (**a**) Gene interaction network of ALYREF-related genes (selected by high confidence 0.900) in STRING. (**b**) Correlation analysis of ALYREF and representative ALYREF-related genes based on GEPIA. (**c**,**d**) Biological process analysis of ALYREF-related genes and ALYREF co-expressed genes. (**e**,**f**) KEGG pathway analysis of ALYREF-related genes and ALYREF co-expressed genes.

To comprehend the functional roles of ALYREF in carcinogenesis, we then turned to investigate the potential pathways that ALYREF involved in. Interacting proteins and most relevant genes of ALYREF were identified for conduction of functional enrichment analysis. Using STRING, we identified 49 ALYREF-interacted proteins with a minimum interaction score of 0.9 and generated a correlation network (Fig. 2a, Table S1). From the above 49 genes, we found that EIF4A3, RBM8A, MAGOH, and SRRT were unanimously positively associated with ALYREF level, and they were involved in mRNA processing, spliceosome assembly, and translation initiation (Fig. 2b). With these findings, we continued to explore the most common functions of these genes correlated with ALYREF. Pan-cancer GO analysis of biological processes indicated that ALYREF-related genes were involved greatly in mRNA processing, RNA splicing, chromosome segregation, organelle fission, and nuclear division (Fig. 2c, d). KEGG analysis revealed that ALYREF-related genes participate in regulation of cell cycle, mRNA surveillance, mismatch repair, and base excision repair in various cancer types (p < 0.01, Fig. 2e, f). The results of KEGG and GO analysis indicated that ALYREF is essential in regulating the cell cycle and mismatch repair in tumor progression.

### Drug sensitivity analysis of ALYREF in cancer

**Figure 3:**
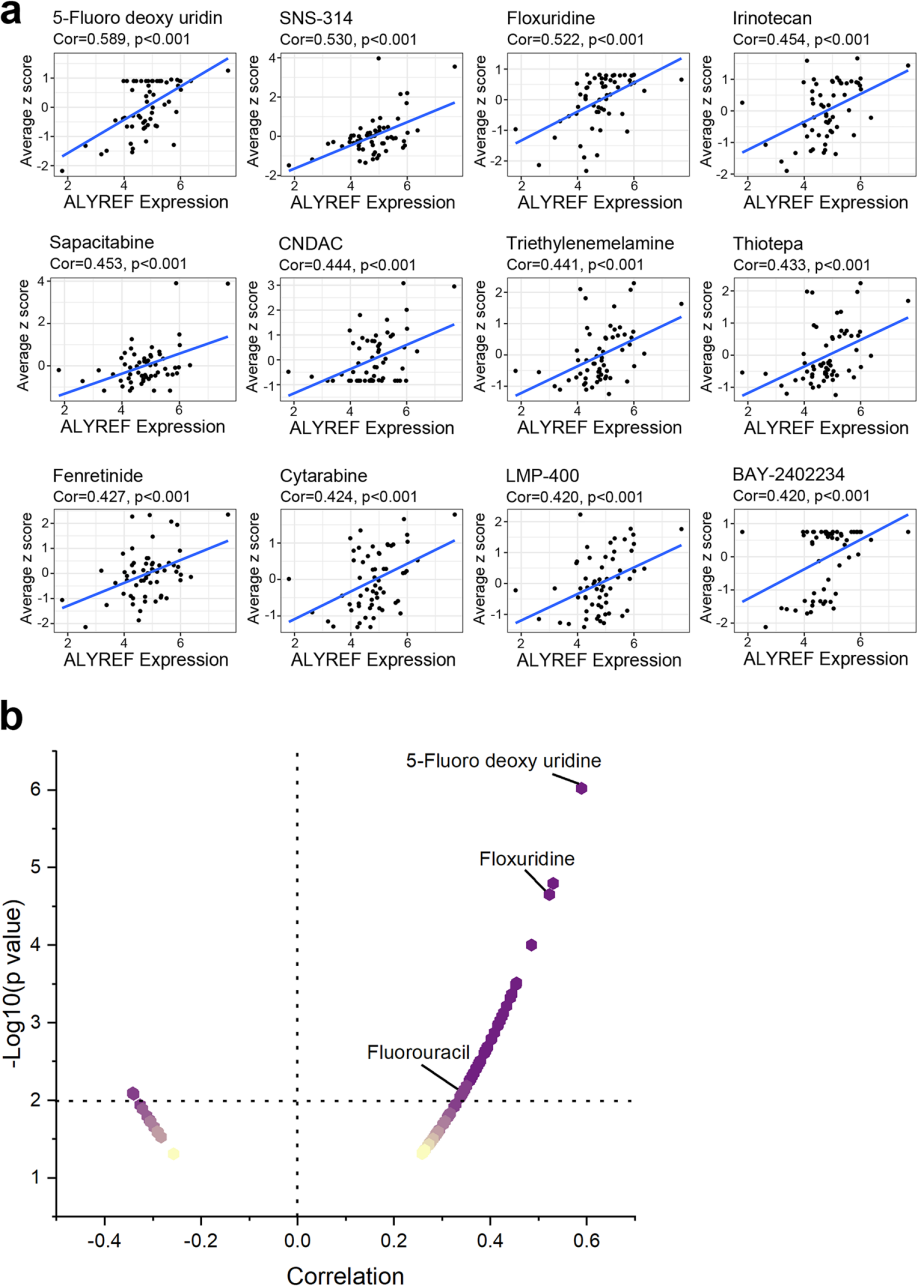
Drug sensitivity testing of ALYREF. (**a**) The correlation of ALYREF expression level and drug sensitivity of different drugs based on NCI-60. (***p-value < 0.001) (**b**) The correlation of ALYREF expression level and drug sensitivity of different drugs based on NCI-60 (*p-value < 0.05) Fluorouracil chemotherapeutic drugs were highlighted.

Due to the importance of ALYREF in tumor development, the relationship between ALYREF expression level and drug sensitivity was studied using CellMiner data. The 12 drugs with the strongest correlations are displayed in Fig. 3 (p < 0.001). The scatter plot exhibited that ALYREF level were observably correlated with the drug sensitivity of fluorouracil chemotherapeutic drugs including 5-Fluoro deoxy uridine, floxuridine, and fluorouracil. The findings implying that patients with high ALYREF level may be better treated with anticancer drugs or associated with resistance to certain chemotherapeutic agents.

### Correlation analysis of genomic heterogeneity of ALYREF gene

**Figure 4:**
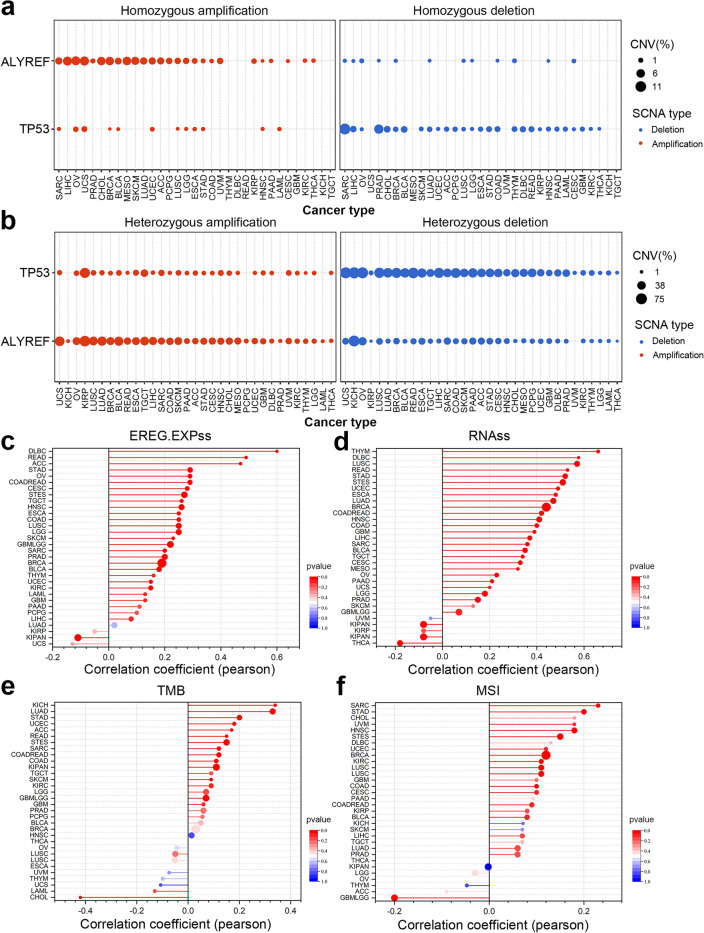
Relationship between ALYREF and genomic heterogeneity and tumor stemness in various cancer types. The profile of homozygous CNV (**a**) and heterozygous CNV (**b**) of ALYREF in various cancer types. Correlation between ALYREF expression level and EREG.EXPss (**c**), RNAss (**d**), TMB (**e**), MSI (**f**) in various cancer types.

To access a more intuitive assessment of ALYREF genomic copy number variations in different cancer types, TP53 (more mutations in many cancers) was set as the reference gene to evaluate the copy number variations (CNVs) of ALYREF. It was showed that ALYREF has more total amplification (including homozygous CNV and heterozygous CNV) in various cancers (Fig. 4a, b). Tumor progression can be greatly stimulated by the acquisition of features resembling those of stem cells. The stemness indices RNAss (Stemness Score based on RNA expression) and EREG.EXPss (Stemness Score based on epigenetically regulated RNA expression) rely on mRNA expression level. In our analysis, ALYREF level were compared to stemness scores among various cancer types. ALYREF was found to be significantly related to EREG.EXPss in 18 cancers (Fig. 4c) and to RNAss in 23 cancers (Fig. 4d), all of which positively related (r > 0.2, p < 0.05). Similar investigations were conducted on tumor heterogeneity. ALYREF expression was significantly related to Tumor mutational burden (TMB) in four cancer types (3 positively related) (Fig. 4e) and to Microsatellite Instability (MSI) in three tumors (2 positively related) (Fig. 4f). Specially, expression level of ALYREF were strongly positively correlated with all four indices in stomach adenocarcinoma (STAD). These results suggested that ALYREF could be specially correlated to the advent of tumor stemness in STAD. Our following studies concentrates on revealing the relationship between ALYREF expression level and STAD tumor situations.

### Expression of ALYREF and prognostic analysis in subtypes of gastric cancer

**Figure 5:**
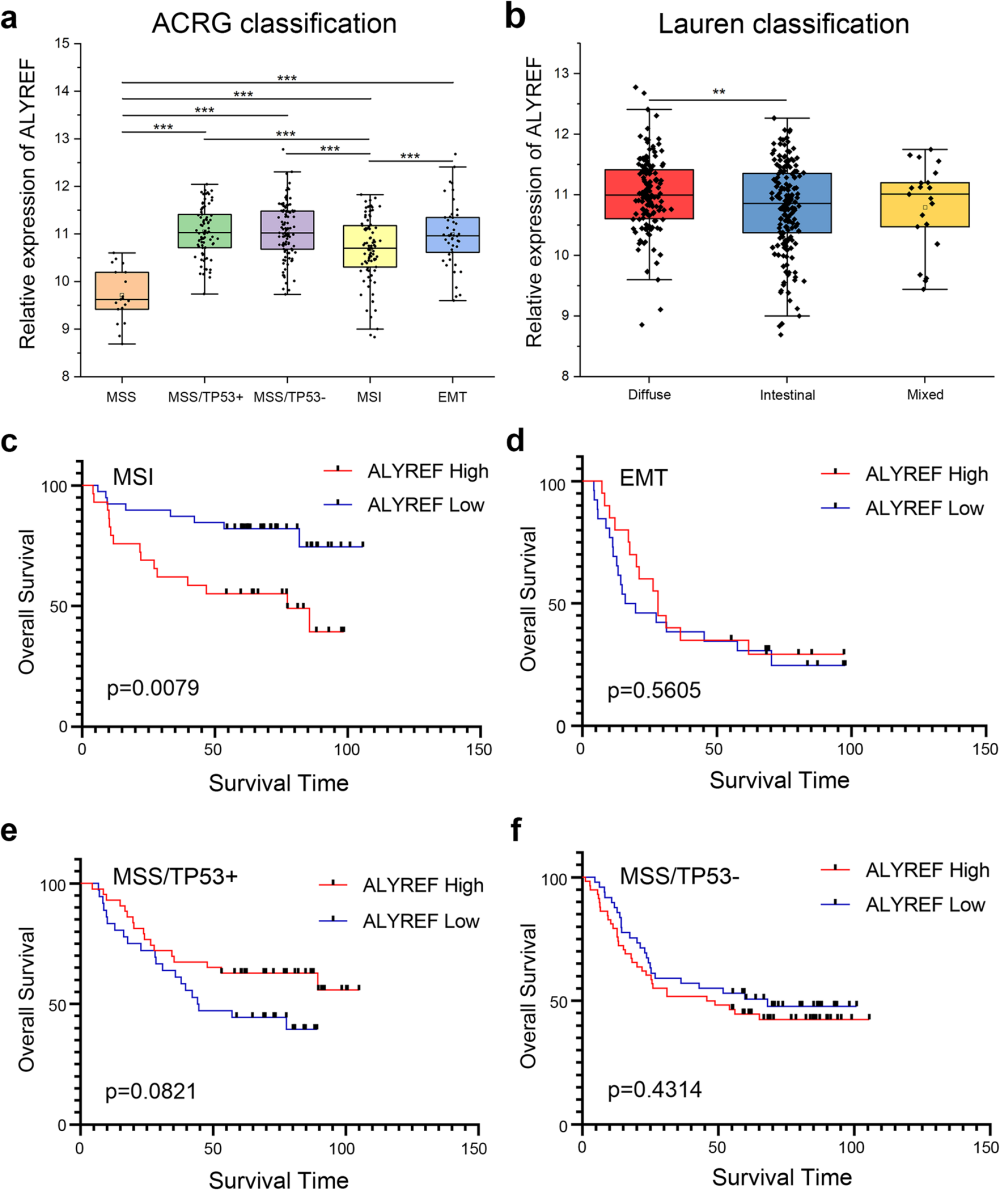
Expression level of ALYREF and survival analysis in different subtypes of STAD. Expression level of ALYREF in different molecular classifications (**a**) and Lauren classifications (**b**) of gastric cancer (**p-value < 0.01, ***p-value < 0.001) (**c**–**f**) Kaplan–Meier analysis of the correlation between ALYREF expression and Overall Survival in different subtypes of gastric cancer. MSI (**c**), EMT (**d**), MSS/TP53+ (**e**), and MSS/TP53− (**f**).

Different classification systems were used to classify gastric cancer. Here we demonstrated the relationship between ALYREF expression level and the different subtypes in two disparate classification systems, including Asian Cancer Research Group (ACRG) classification and Lauren classification. Based on ACRG classification, ALYREF was observably low expressed in microsatellite stability (MSS) patients (p < 0.001, Fig. 5a). Under Lauren classification, ALYREF highly expressed in diffuse-type gastric cancer compared with intestinal-type gastric cancer (p < 0.01, Fig. 5b). Furthermore, prognostic analysis of different molecular subtypes of gastric cancer unfolded that high ALYREF expression in MSI GC patients leading to poor prognosis (p < 0.01, Fig. 5c). However, in other subtypes of GC, abnormal expression level of ALYREF had no significant effect on prognosis in GC patients (Fig. 5d–f).

### Enrichment analysis of ALYREF-related genes in gastric cancer

**Figure 6:**
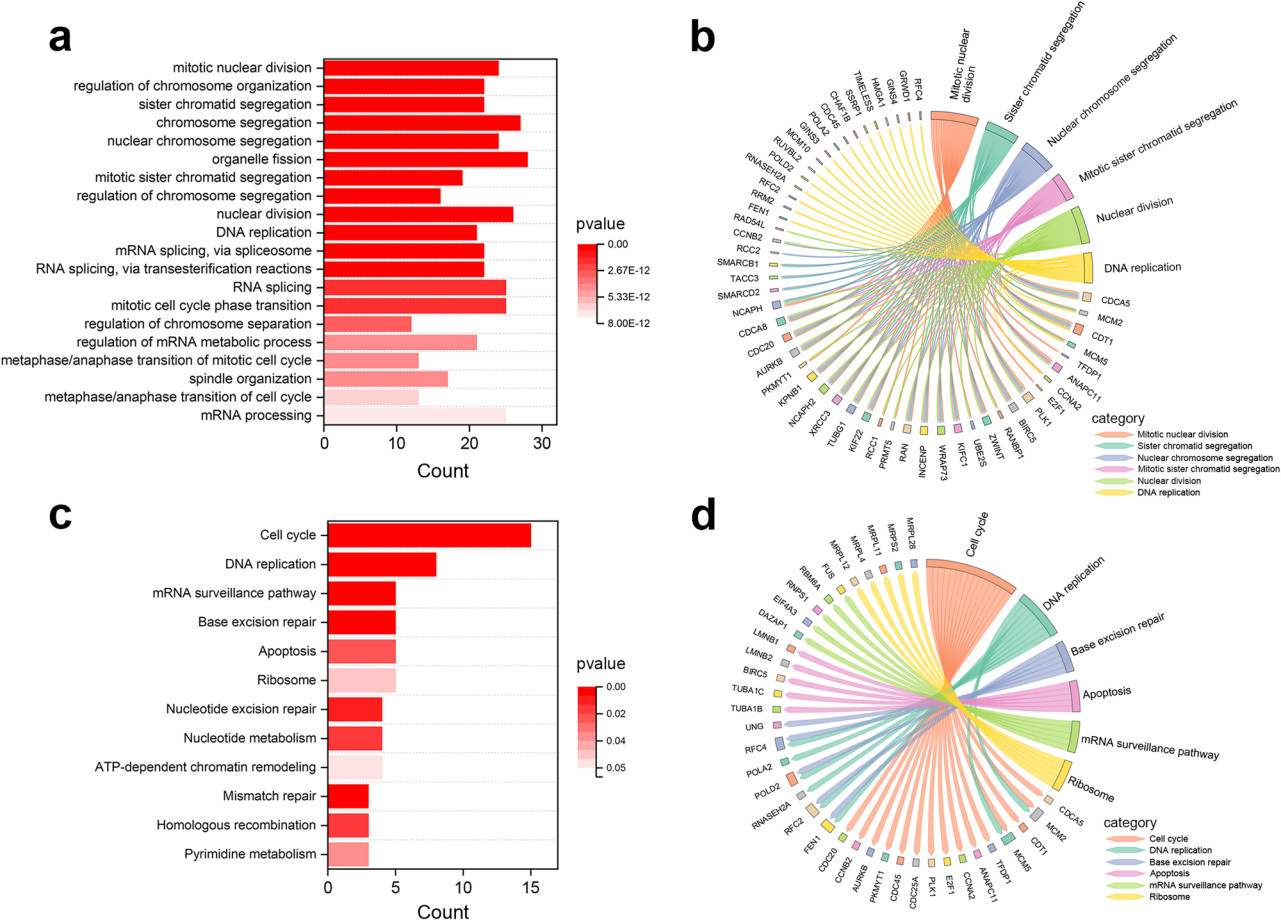
Biological function analysis of ALYREF co-expressed genes in STAD. (**a**,**b**) Biological process analysis of ALYREF co-expressed genes in gastric cancer. (**c**,**d**) KEGG pathway analysis of ALYREF co-expressed genes in gastric cancer.

Next, we moved to the enrichment factors of ALYREF-related genes in gastric cancer. GO analysis of biological processes indicated that these genes were involved greatly in chromosome segregation, nuclear division, and organelle division (Fig. 6a, b). KEGG enrichment analysis showed that 15 of these related genes were correlated with the cell cycle. Base excision repair, apoptosis, mRNA surveillance pathway, and DNA replication were also common functions related to at least 5 genes with a significant correlation (p < 0.05) (Fig. 6c, d). And then, we conducted biological experiments to reveal the actual effect of ALYREF on gastric cancer cells.

### ALYREF plays an important role in cell proliferation of GC

**Figure 7:**
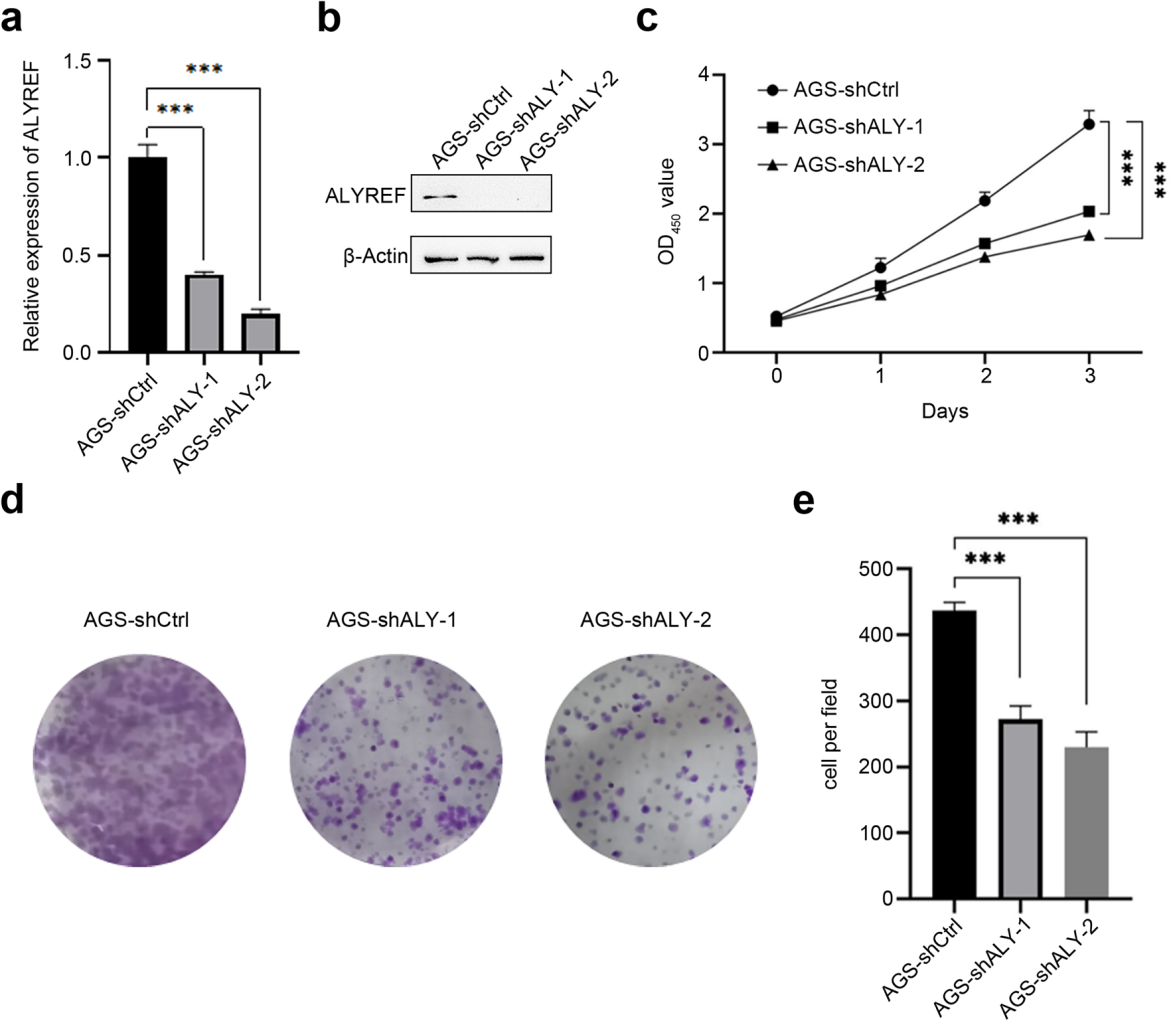
Effect of ALYREF on proliferation of gastric cells. (**a**,**b**) Verification of shRNA-mediated knock-down efficiency of ALYREF in AGS cells on mRNA level and protein level. The protein samples were derived from the same experiment. (**c**) Cell viability of AGS cells after transfection by using CCK-8 assay. (**d**,**e**) Colony formation assay in AGS cells under control conditions or after ALYREF knock-down (***p-value < 0.001).

**Figure 8:**
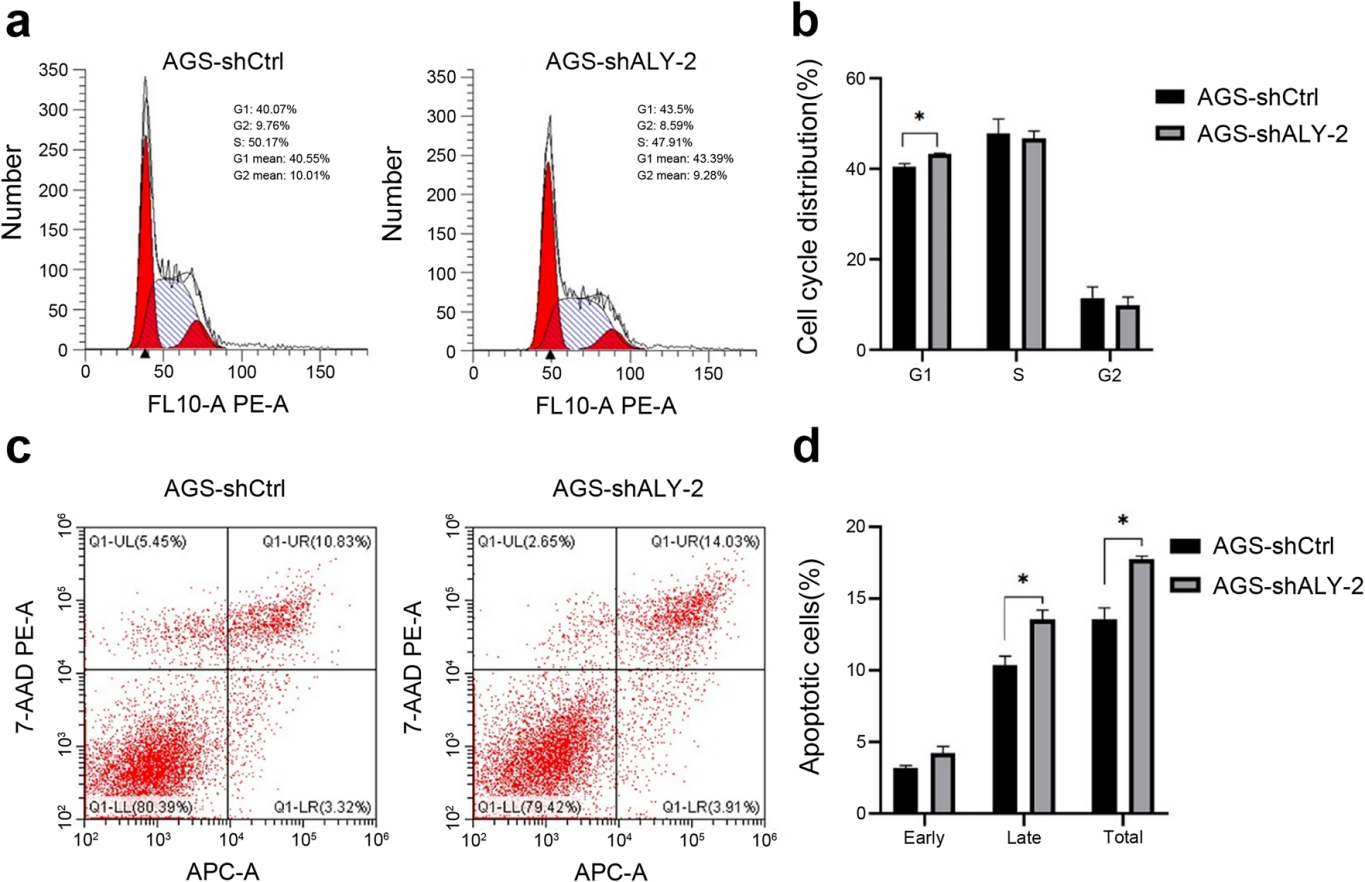
Effect of ALYREF on cell cycle and apoptosis. (**a**,**b**) Cell cycle of AGS cells was analyzed by flow cytometry. (**c**,**d**) Apoptosis rate of AGS cells under control conditions or after ALYREF knock-down (*p-value < 0.05).

The mRNA expression of ALYREF in AGS shALY (shALYREF) and shCtrl cells was detected by RT-qPCR. RT-qPCR verified that shALY-1 and shALY-2 both effectively knocked down the expression level of ALYREF compared with shCtrl (Fig. 7a). Western Blot assay also confirmed the knockdown efficiency of shALY-1 and shALY-2 in GC cells (Fig. 7b, Fig. S1). The sequences of shRNA were listed in methods and materials. CCK-8 assay displayed that shALY-1 and shALY-2 decreased cell proliferation ability (Fig. 7c). Besides, after knocking down the expression of ALYREF, the number of cell clone foci in shALY-1 and shALY-2 was significantly less than that in shCtrl, indicating that the clone formation ability of the cells in the knockdown group was significantly weakened (Fig. 7d, e). To further confirm the biological functions of ALYREF in gastric cancer cells, flow cytometry was employed to detect the portion of cells in different cell cycle and apoptosis. The data showed that knocking down of ALYREF contributed to cell cycle arrest in G1 phase (Fig. 8a, b). Apart from that, the amount of non-viable apoptotic cell also increased after knocking down of ALYREF (Fig. 8c, d). These results substantiated the deductions we came up with above that ALYREF plays a key role in regulating the cell cycle and cell apoptosis in gastric cancer.

## Discussion

ALYREF, also known as ALY, REF, BEF, and THOC4, was identified by researchers’ investigation of RNA synthesis and RNA nuclear export. After RNA splicing, to guarantee the spliced mRNA to leave the nucleus instead of the pre-mRNA, a special form of RNA–protein complex was assumed to fulfill this progress^16^. Further studies revealed that ALYREF is recruited during spliceosome assembly and enhances both the speed and efficiency of mRNA export in vitro^11,17^. It was confirmed that mRNA export is correlated with of ALYREF level in yeast^18,19^. Another research examined the interaction between ALYREF and the conserved DEAD-box helicase UAP56, and showed both them presented concurrently in mRNP. In Xenopus oocytes nuclei and HeLa nuclear extract, ALYREF combined tightly with UAP56 in the spliced mRNP, which enhanced the export efficiency of mRNA^10^. Besides, ALYREF was revealed to facilitate DNA binding by recognizing the unfolded leucine zipper and expediting the folding process of bZIP monomers into dimers^20^. During RNA transcription, ALYREF can increase DNA binding by both LEF-1 and AML proteins, thus promoting the transcription in the context of the TCR alpha enhancer in Hela cell^21^. These evidences manifested that ALYREF is a special class of molecular chaperone, which regulates the activity of nuclear transcription factors^20^.

ALYREF was clarified to be a recognition protein of 5-methylcytosine (m5C)^9^, while m5C is a universal modification identified in mRNA, tRNA, rRNA, ncRNA, and it has been demonstrated to regulate multiple biological process^22^. It has been indicated that m5C modification on RNA is essential for the migration and differentiation of stem cells^23,24^. Additionally, m5C has been identified as a critical molecular event adjusting mRNA stability during early embryogenesis across vertebrates^25^. Regulatory factors of m5C have also been implicated to be involved in carcinogenesis by affecting cell migration and metastasis^5^. Members of the NOL1/NOP2/SUN domain (NSUN) family (NSUN1–7) and tRNA aspartic acid methyltransferase 1 (TRDMT1, also known as DNMT2) act as ‘writers’ by adding m5C modification, while NSUN2 and NSUN6 are the specific methyltransferases in mRNA^26,27^. ALYREF functions as the “reader”, binding to m5C site on mRNA and either enhancing the stability or accelerating the nuclear export of mRNA^28^.

Some studies showed that ALYREF play a role in the initiation and progression of tumors. Knockdown of ALYREF significantly suppressed cell growth and increased the rate of apoptosis in hepatocellular carcinoma (HCC) cells in vitro and in vivo^8^. In bladder cancer (BCa), ALYREF stabilized PKM2 mRNA by binding to its m5C sites, promoting the proliferation of BCa cells through PKM2-mediated glycolysis^29^. Additionally, ALYREF contributes to the malignancy of urothelial carcinoma of the bladder (UCB) by maintaining the stability of RABL6 and TK1 mRNA^30^. High expression level of ALYREF has been shown to be a significant factor in poor survival in breast cancer patients, and facilitated the tumorigenesis of breast cancer cells in mice^31^. Meanwhile, ALYREF was suggested to function as an oncogenic factor in non-small cell lung cancer (NSCLC) by stabilizing LINC02159^32^. ALYREF has been found to contribute to the malignant phenotype of lung adenocarcinoma (LUAD) by stabilizing YAP^33^. ALYREF facilitated the stabilization of MYCN, which drive cancer cells malignant in neuroblastoma (NB)^34^. Multi-Omic Analyses and a novel prediction model have also demonstrated a significant correlation between ALYREF and both tumor-node-metastasis stages and poor prognosis in HCC and Ovarian cancer (OC)^15,35^.

Based on the current pan-cancer analysis, ALYREF may be widespread involved in carcinogenesis in various cancers, including the regulation of cell cycle and apoptosis. This hypothesis was further supported by RNA immunoprecipitation (RIP) experiment, which revealed that the genes targeted by ALYREF were involved in pathways related to cell cycle and necroptosis^8^. Association between ALYREF and drug sensitivity was established through the analysis of NCI-60 cell lines, demonstrating a significant correlation between ALYREF and fluorouracil drugs. It has been reported that adverse effects were observed in chemotherapy whether using 5-FU combined with oxaliplatin or 5-FU alone^36,37^. For the relationship between fluorouracil drugs and cell cycle inhibition^38^, this study implied function of ALYREF on cell cycle provides a potential mechanism of ALYREF impacting on the sensitivity to fluorouracil drugs.

According to these four important indicators (TMB, MSI, RNAss, and EREG.EXPss) associated with tumorigenesis and development, STAD stands out as the only type of cancer that was significantly associated with ALYREF across these dimensions. Notably, prognostic analysis in different STAD subtypes revealed a significant positive correlation between elevated ALYREF level and poor prognosis specifically in MSI-high STAD. MSI is defined as a hyper-mutable phenotype that occurs at genomic MS in the presence of deficient DNA mismatch repair (dMMR) machinery. Finding an effective remedy for MSI-high STAD remains challenging due to its high mutation rate and the molecular and phenotypical heterogeneity of STAD^39,40^. An immunotherapy-based meta-analysis also showed that patients with MSI-high STAD were highly immunosensitive^41^. As a result, European Society for Medical Oncology (ESMO) has established distinctive therapeutic regimens for MSI-high STAD patients^42^. In STAD, the co-expressed genes of ALYREF are enriched in base excision repair, mRNA surveillance pathway, nucleotide excision repair, and mismatch repair, indicating the crucial role of ALYREF in MSI-high STAD. 5-fluorouracil has shown to be more beneficial in dMMR/MSI-H STAD patients compared with STAD patients with mismatch repair protein deficiency (MMRD)^43^. However, there are also studies claiming that the adjuvant therapy of fluorouracil was ineffective on dMMR patients^44^. Further exploration of the function of ALYREF in STAD is absolutely necessary for clarifying the kind of role it plays in tumor heterogeneity and MSI in STAD.

GO and KEGG analysis revealed that ALYREF-related genes enriched in the regulation of cell cycle and cell apoptosis, which further impacts the progression of GC tumor. Subsequently, we conducted in vitro function experiments to validate the results, demonstrating that knockdown of ALYREF inhibited cell proliferation, arrested cell cycle and induced cell apoptosis in the gastric cancer cell line AGS.

## Conclusions

To our knowledge, this is the first comprehensive pan-cancer analysis on oncogenic function of ALYREF in tumors. We demonstrated that the expression of ALYREF was significantly increased in the tumor tissues of pan-cancer and was associated with clinical poor prognosis. Our study also identified that ALYREF was may be involved in the occurrence and progression of STAD by regulating of cell cycle and cell apoptosis. It may provide commonly therapeutic methods for tumors with high level of ALYREF expression.

## Methods and materials

### Differential expression of ALYREF and prognostic value in pan-cancer analysis

The data of ALYREF and related clinical information of 33 common cancer types were downloaded from the Cancer Genome Atlas (TCGA, https://portal.gdc.cancer.gov/). The data of ALYREF expression in normal tissues were obtained from genotype-tissue expression database (GTEx, http://commonfund.nih.gov/GTEx/). Both TCGA and GTEx datasets were carried out comparisons of ALYREF expression between cancerous and adjacent normal tissues. Transcripts per million (TPM) was used to normalize the gene expression level among different samples. Distributions of gene expression level are displayed using box plots. The statistical significance calculated by the Wilcoxon test is represented by the number of stars in the figures (one: p-value < 0.05; two: p-value < 0.01; three: p-value < 0.001). The prognostic value of ALYREF in various cancer types were evaluated by univariate Cox analysis and Kaplan–Meier analysis based on TCGA and GTEx database. In addition, the relationship between the expression level of ALYREF and different cancer stages were investigated using the “Clinical stage and gene expression analysis” function.

### Enrichment analysis of ALYREF-related genes in pan-cancer

The STRING database (https://cn.string-db.org/) was used to find proteins interacting with ALYREF whose interaction scores were limited at 0.900^45^. An interacting network was plotted to show the correlations between ALYREF and the selected interacting genes. Then, GEPIA2 (“Similar Genes Detection” function) was used to screen for the top 200 genes possessing a similar expression pattern with the ALYREF gene in various cancer. These similar expression pattern genes and interacting genes were put into the DAVID database (https://david.ncifcrf.gov/) to explore their relationship with various biological processes through Gene ontology (GO) and Kyoto encyclopedia of genes and genome (KEGG) enrichment analyses^46^. P value < 0.05 was considered to be statistically significant. The “Correlation Analysis” function of GEPIA2 was used to calculate the correlation between ALYREF and genes selected from STRING in multiple cancer types. Pearson Correlation Coefficient was computed by the non-log scale for plotting, and the log-scale axis for visualization.

### Drug sensitivity analysis of ALYREF

Gene expression data and drug sensitivity data were downloaded from the CellMiner (https://discover.nci.nih.gov/cellminer/). CellMiner is an open available database normalized for multiple molecular feathers including RNA, protein, and pharmacological level, which is listed 60 cancer cell types by the National Cancer Institute Cancer Research Center (NCI). Cell sensitivity to a certain type of drug was represented by the z-score obtained in the compound activity profile. Higher z-score value indicates increased anticancer activity. R software was used to analyze the drug sensitivity data using Pearson’s correlation.

### Correlation between ALYREF level and tumor stemness

The GDC database (https://portal.gdc.cancer.gov/) was used to assess the relationships between ALYREF level and tumor stemness and heterogeneity among TCGA tumor specimens by conducting Pearson’s correlation analysis. RNA data were used to compute different stemness scores including RNA expression-based (RNAss) and epigenetically regulated RNA expression-based (EREG.EXPss). Tumor heterogeneity was assessed using indices like Tumor Mutation Burden (TMB) and Microsatellite Instability (MSI) from TCGA RNA-seq data. The correlation between these indexes and ALYREF level was evaluated by the Pearson correlation coefficient (r) that ranges from − 1 to 1. Absolute value greater than 0.2 were viewed as the indication of a strong correlation. In addition, GSCALite was employed to explore the relationship between ALYREF level and copy number alterations (CNA) in various cancer types.

### Expression of ALYREF and prognostic analysis in different subtypes of STAD

GENT2 (http://gent2.appex.kr/gent2/) was used to explore the expression level of ALYREF in different subtypes of STAD. The prognostic analysis of different molecular classifications of gastric cancer was employed based on database gained from GENT2.

### Cell culture and transfected with shRNA of ALYREF and its control

The human gastric cell line AGS was purchased from Shanghai Cell Bank, Chinese Academy of Sciences. PRMI 1640 culture medium (Nanjing Vicente Biological Company) supplemented with 10% fetal bovine serum (FBS) was used to culture cells. Cells in good condition were observed to be transfected with lentivirus (ALYREF shRNA#1: 5ʹ-GCCGATATTCAGGAACTCTTT-3ʹ; ALYREF shRNA#2: 5ʹ-GCGTAAACAGAGGTGGCATGA-3ʹ).

### RNA extraction and Real-time quantitative PCR (qPCR)

Total RNA was extracted from cells using TRIzol reagent (Invitrogen) according to the manufacturer’s instructions. Q-PCR was performed with a SYBR Premix Ex Taq Kit (Takara, Japan) and a StepOne Plus system (Applied Biosystems, Foster City, CA) in triplicate. The relative level of ALYREF were normalized by β-actin. All data were calculated using the 2^−ΔΔCt^ method. The primers sequences were: β-actin forward, 5ʹ-GTCATTCCAAATATGAGATGCGT-3ʹ; β-actin reverse, 5ʹ- GCTATCACCTCCCCTGTGTG-3ʹ; ALYREF forward, 5ʹ- TCTGGTCGCAGCTTAGGAAC-3ʹ; ALYREF reverse, 5ʹ- TGCCACCTCTGTTTACGCTC-3ʹ.

### Western blot

AGS cells were lysed with RIPA lysis buffer (Yeasen, 20101ES60) on ice, and the supernatant were collected for preparing protein samples. SDS-PAGE gels were used for electrophoresis, and separated proteins were blotted on PVDF membrane. After being blocked for 1.5 h at room temperature, the layer was brooded with primary antibodies anti-ALYREF (Santa Cruz, sc-32311), anti-β-Actin (Sigma, A5441) overnight at 4 ℃. HRP-labeled anti-mouse antibodies (Cell Signaling Technology, 7076) were used for detection of the primary antibodies. After being treated with ECL luminescent solution (Vazyme, E423-01), images were acquired using Chemiluminescence scanners (Tanon).

### Cell proliferation assay

After transfected with shALY and its control for stable, cell viability was detected by the cell counting kit-8 (CCK-8) assay kits (Vazyme Biotech Co., Ltd, Cat: A311-02-AA). Colony formation assay was employed to monitored long-term cell survival. Ater 1000 cells were seeded into 6-well plates and grown for 2 weeks, the cells were fixed with 4% paraformaldehyde and visualized by 0.5% (w/v) crystal violet (Sigma-Aldrich) dyeing. In every experiment, knock-down efficiency was tested in cells simultaneously cultured to assure the exactitude of experimental results. Graph Pad Prism 8 was applicated to conduct statistical analysis and complete the charts.

### Flow cytometry analyze cell cycle and apoptosis

Synchronized gastric cancer cells AGS with shALY or Control were dissociated by 0.25% pancreatic enzyme without EDTA. Isolated cells were washed through PBS buffer solution and then resuspended in 70% ethanol. Processed cells were detected by flow cytometry to calculate the proportion of cells in different cell cycles. Apoptosis was measured by Annexin V-fluorescein isothiocyanate (FITC) apoptosis detection kit (KeyGEN BioTECH, Nanjing, China) according to the manufacturer's instructions.

### Supplementary Information

Supplementary Table S1.

Supplementary Figure S1.

## Data Availability

The datasets used to investigate the research are available in online databases. These datasets can be found in the Cancer Genome Atlas (TCGA, https://portal.gdc.cancer.gov/), genotype-tissue expression database (GTEx, http://commonfund.nih.gov/GTEx/), and the CellMiner (https://discover.nci.nih.gov/cellminer/).
