# Supplementary figures and images for: Identification of ALYREF in pan cancer as a novel cancer prognostic biomarker and potential regulatory mechanism in gastric cancer
Source: Sci Rep. 2024 Mar 15;14:6270. doi: 10.1038/s41598-024-56895-5 (PMC10942997; doi:10.1038/s41598-024-56895-5)

**a**

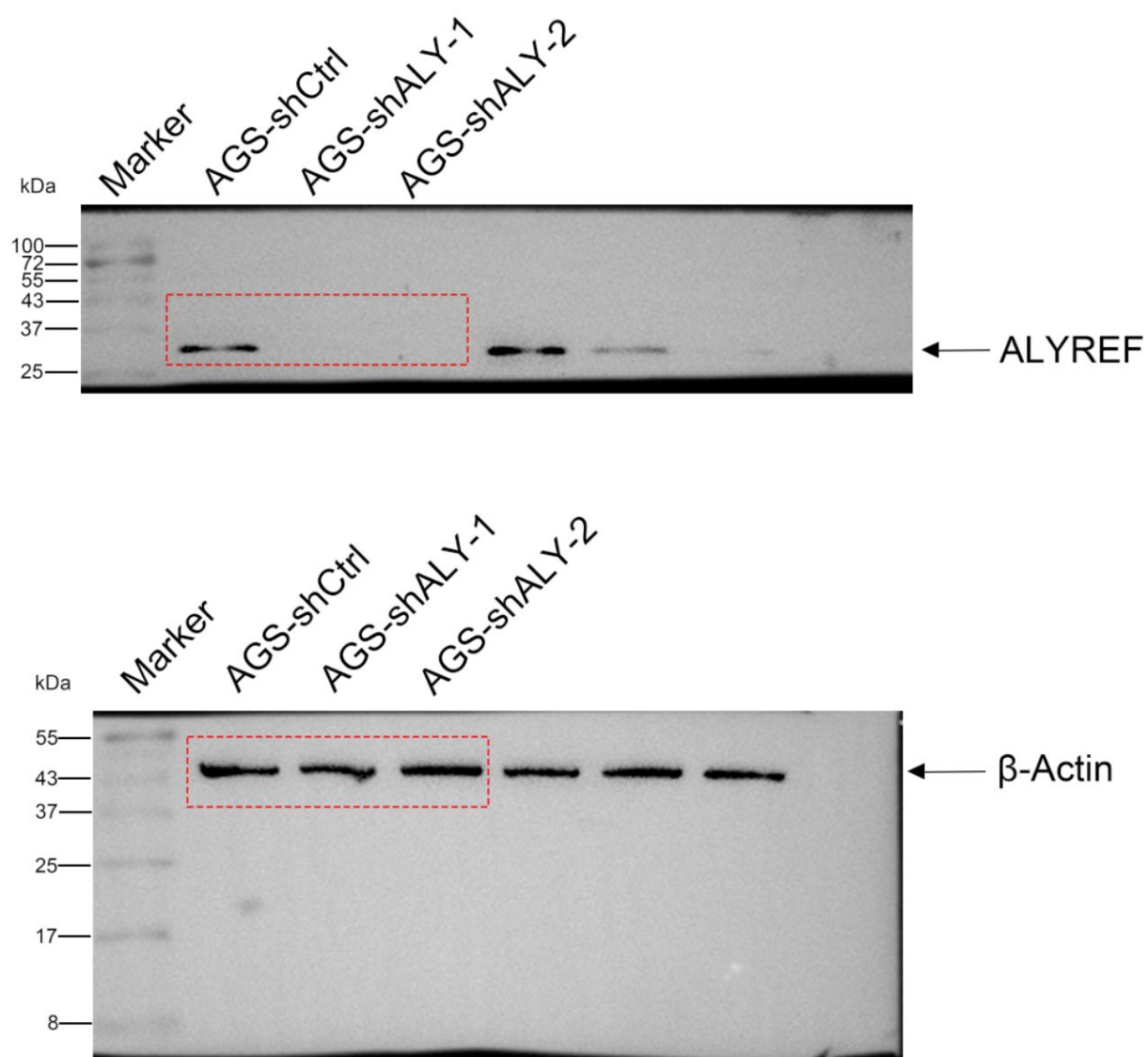

Fig. S1. Relative ALYREF expression in gastric cancer cells detected by Western Blot assay.

Supplement: Supplementary file 2 — Supplementary Figure S1. [file 41598_2024_56895_MOESM2_ESM.pdf]
